# Development of an innovative *in vivo* model of PJI treated with DAIR

**DOI:** 10.3389/fmed.2022.984814

**Published:** 2022-10-13

**Authors:** Hervé Poilvache, Françoise Van Bambeke, Olivier Cornu

**Affiliations:** ^1^Neuro Musculo-Skeletal Laboratory, Institut de Recherche Expérimentale et Clinique, Université catholique de Louvain, Brussels, Belgium; ^2^Cellular and Molecular Pharmacology Laboratory, Louvain Drug Research Institute, Université catholique de Louvain, Brussels, Belgium; ^3^Orthopedic Surgery and Traumatology Department, Cliniques universitaires Saint-Luc, Brussels, Belgium

**Keywords:** prosthetic joint infection, *in vivo*, DAIR, vancomycin, pharmacokinetics, rabbit, knee

## Abstract

**Introduction:**

Prosthetic Joint Infection (PJI) are catastrophic complications of joint replacement. Debridement, implant retention, and antibiotic therapy (DAIR) is the usual strategy in acute infections but fails in 45% of MRSA infections. We describe the development of a model of infected arthroplasty in rabbits, treated with debridement and a course of vancomycin with clinically relevant dosage.

**Materials and methods:**

A total of 15 rabbits were assigned to three groups: vancomycin pharmacokinetics (A), infection (B), and DAIR (C). All groups received a tibial arthroplasty using a Ti-6Al-4V implant. Groups B and C were infected per-operatively with a 5.5 log10 MRSA inoculum. After 1 week, groups C infected knees were surgically debrided. Groups A and C received 1 week of vancomycin. Pharmacokinetic profiles were obtained in group A following 1st and 5th injections. Animals were euthanized 2 weeks after the arthroplasty. Implants and tissue samples were processed for bacterial counts and histology.

**Results:**

Average vancomycin AUC_0–12 h_ were 213.0 mg*h/L (1st injection) and 207.8 mg*h/L (5th injection), reaching clinical targets. All inoculated animals were infected. CFUs were reproducible in groups B. A sharp decrease in CFU was observed in groups C. Serum markers and leukocytes counts increased significantly in infected groups.

**Conclusion:**

We developed a reproducible rabbit model of PJI treated with DAIR, using vancomycin at clinically relevant concentrations.

## Introduction

Prosthetic Joint Infections (PJIs) are a catastrophic complication of joint replacement surgery (1) and are one of the leading causes of failure following hip and knee replacement (2, 3). PJIs and their treatments generate significant morbidity (4) and mortality (5) in affected patients and represent an important burden to healthcare systems (6–8).

PJIs are caused by a variety of pathogens. *Staphylococcus aureus* is the most frequently isolated species (9, 10). Among these, Methicillin-Resistant *S. aureus* (MRSA) strains represent 12.8–48.1% of isolates, with important variations between countries (11). MRSA strains are of particular concern in the setting of PJIs as these strains are highly prevalent in acute infections (12) and are a significant risk factor for treatment failure following debridement surgery, implant retention, and antibiotic therapy (DAIR) (13). DAIR is usually favored in acute infection cases – occurring less than 4 weeks after the index surgery – as it is a less invasive procedure with superior functional outcomes compared to staged implant replacement (14, 15). However, patients suffering from an MRSA PJI suffered from a substantial failure rate in a large multi-centric study (13).

This high failure rate is multifactorial but is in part due to the propensity of *S. aureus* to attach to surfaces or aggregate and form biofilms (16). Biofilms are bacterial communities embedded in a complex self-produced extracellular matrix that isolates them from their environment (17). Bacteria in biofilms tolerate antibiotic concentrations up to 1000-fold higher than planktonic bacteria (18) and can evade the host’s immune system (19), leading to chronic infections.

Consequently, the development of biofilm targeting therapies with a potential application in orthopedics is of particular interest (20). Multiple strategies and therapeutic agents have been described, including quorum sensing inhibitors (21), antibacterial peptides (22), physical disruption of biofilm (23), and enzymatic digestion of the extracellular matrix (24). We recently reported promising *in vitro* results using a broad-spectrum tri-enzymatic cocktail (TEC) in combination with multiple classes of antibiotics against MRSA, MRSE, and *E. coli* biofilms (25) leading us to design a clinically relevant PJI *in vivo* model adequate for testing innovative therapeutic strategies in the setting of DAIR strategies.

Multiple *in vivo* models have been proposed in the literature to study PJI and its treatment. These vary in complexity but for the most part poorly reproduce the periprosthetic environment, limiting their clinical relevancy [(26) for review].

The objective of this study is the development of a clinically relevant model of knee arthroplasty in New Zealand White (NZW) rabbits, using Ti-6Al-4V 3D printed unicompartmental tibial implants, infected with an MRSA reference strain. The development of this model integrates the study of vancomycin pharmacokinetics in NZW rabbits to reproduce the clinical targets used in the treatment of *S. aureus* infections. The impact of DAIR on the bacterial load of the implant and relevant tissues, inflammatory markers, and histological appearance of joint tissues were compared to control animals.

## Materials and methods

### Study design

**FIGURE 1:**
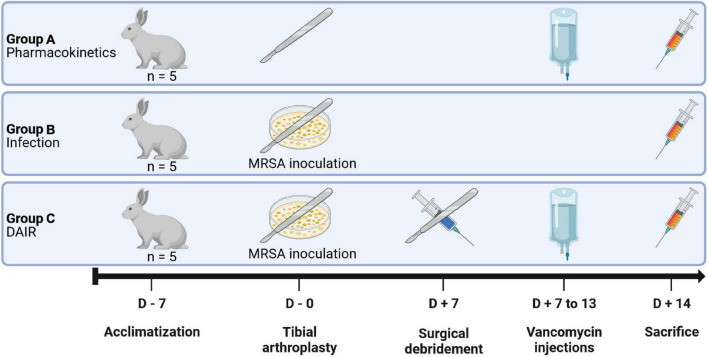
Experimental design. The animals were assigned to three groups of five animals each. Group A animals were used to confirm the validity of the implants designs and to assay the pharmacokinetics of vancomycin; Group B animals were used to confirm the adequacy of the bacterial inoculum; Group C animals were infected and treated with a surgical debridement followed by the intra-articular injection of Tris-HCl buffer and a 1 week course of IV vancomycin.

*In vivo* experiments were performed following the approval of the protocols by the local ethics committee (2019/UCL/MD/041). A total of 15 female New-Zealand White (NZW) rabbits were assigned to three groups in a non-blinded way: group A: vancomycin pharmacokinetics, group B: infection, group C: DAIR treatment (Figure 1). All animals benefitted from a minimum of 7 days of acclimatization period during which no treatment was performed. After the acclimatization period, a unicompartmental tibial arthroplasty was performed in all groups with a custom-designed Ti-6Al-4V implant. The joints of the animals from groups B and C were inoculated with 5.5 log10 CFU of MRSA strain ATCC 33591. After 7 days, the infected joints of the animals of groups C were surgically debrided. Animals of groups A and C received IV injections of 50 mg/kg of vancomycin twice a day 7 days after the initial surgery (group A) or immediately after the debridement procedure (groups C). In group A animals, pharmacokinetic profiles of serum vancomycin were performed following the 1st and 5th injections. Blood samples were obtained every 2 days for total leucocyte counts, differential leukocyte counts, serum CRP and procalcitonin concentrations measurements, and serum vancomycin monitoring (groups C). All animals were sacrificed 14 days after the initial surgery. The implants and samples from the joint tissues were processed for microbiological assessment and histology.

### Animals

A total of 15 specific pathogens-free female NZW rabbits weighing 3.0–3.5 kg were obtained from CER Groupe (Marloie, Belgium). The animals were held in a level 2 biosafety room, with controlled temperature and hygrometry and automated lightning set to a 12 h/12 h regimen. The animals were kept in cages with a ground surface of 6625 cm^2^ and a height of 51 cm, equipped with a raised platform and a shelter, alone or in pairs. The cages’ floors were covered with wood chippings and cleaned daily. The animals were watered *ad libitum* with fresh water and were fed *ad libitum* with hay and rabbit-specific pellets. The environment was further enriched with wooden toys.

The humane endpoint was defined by a weight loss of over 20% over the experimental period of 2 weeks, wound opening, or if the animals exhibited signs of systemic infection such as shaking, cyanosis, ataxia.

### Bacteria

The Methicillin-Resistant *Staphylococcus aureus* strain ATCC 33591, previously used in our laboratory for biofilms models, was used. The MIC (1 μg/ml) and MBEC (> 1024 μg/ml) for vancomycin were determined previously (25). Bacteria were grown overnight on tryptic soy agar (TSA) plates from frozen stock. Some colonies were then suspended in sterile PBS, adjusted to an OD_620 nm_ of 0.5 (∼8 log_10_ CFU), and diluted 1:10 in PBS. The bacterial suspension was stored at 4°C until inoculation (1.5–2 h). Serial dilutions of the bacterial inoculum were plated on TSA to confirm the bacterial inoculum.

### Antibiotics

Vancomycin powder for injection (Mylan bvba, Hoeilaart, Belgium) was reconstituted with 0.9% saline (Baxter SA, Lessines, Belgium) at a 50 mg/ml concentration.

### Implant design

**FIGURE 2:**
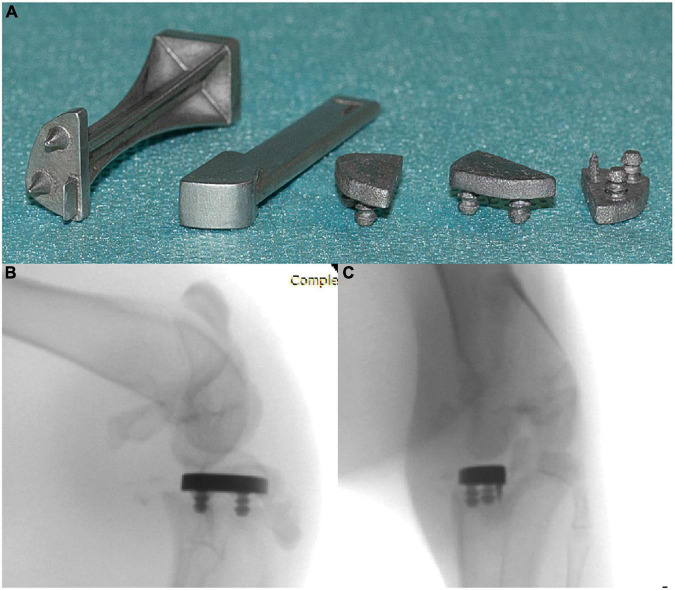
**(A)** Ti-6Al-4V implants (right) and instruments (left). Post-mortem radiographs showing the position of the implant in AP **(B)** and lateral **(C)** views.

Uni-compartmental tibial implants were designed by the authors using Fusion 360 (Autodesk, CA, USA) following the dissection of the knees 4 NZW rabbits carcasses provided by CER Groupe (Marloie, Belgium). The final implant design and specific instruments were 3D printed in Ti-6Al-4V by Materialise NV (Leuven, Belgium) (Figure 2).

### Procedures

#### Anesthesia and analgesia

General anesthesia was obtained by the intramuscular injection of 35 mg/kg of ketamine (Nimatek, Dechra Pharmaceuticals PLC, Northwich, UK), 5 mg/kg of xylazine (Rompun, Bayer AG, Leverkusen, Germany), and 0.1 mg/kg of butorphanol (Dolorex, MSD Animal Health, Kenilworth, NJ, USA). Anesthesia was confirmed by the loss of muscle tone and the absence of corneal reflex. The cornea was protected by the application of an ocular gel (Ocugel, URSAPHARM GmbH, Saarbrücken, Germany). The rabbits received an IV perfusion of 800 mg of paracetamol (Fresenius Kabi, Bad Homburg vor der Höhe, Germany) during the surgery. A total of 12.5 μg/h transdermal fentanyl patches (Durogesic, Janssen-Cilag NV, Beerse, Belgium) were applied immediately after the surgery for post-operative analgesic treatment.

#### Arthroplasty

The hairs of the operated leg were trimmed on the eve of the surgery. Following general anesthesia, the animals were installed in a Class II biosafety cabinet. The operated leg was disinfected twice with 2% chlorhexidine gluconate alcohol solution (Qualiphar, Bornem, Belgium) and the leg was sterile draped. The joint was approached through a medial parapatellar incision. The medial meniscus was resected and 5 mm of the medial tibial plateau was removed using a high-speed burr. The cut height was confirmed using a spacer block. The bone surface was then further prepared using a custom tibial punch. After irrigation of the joint space with sterile saline, the final implants were impacted. The joint was then further irrigated with sterile saline. The joint capsule, subcutaneous tissues, and skin were closed separately with running sutures using a 3/0 polyester thread. The joints of groups B and C animals were inoculated by intra-articular injection of 100 μl of a 6.5 log_10_ CFU/ml MRSA suspension after the closure of the subcutaneous tissues. The bacterial inoculum was selected to reflect the usual range of inoculum used in the literature [e.g., (27–30)]. The wounds were then covered using an antiseptic spray and dressed.

#### Debridement

Animals of groups C were operated on 1 week after the initial surgery. Following anesthesia, the operated leg was disinfected twice with 2% chlorhexidine gluconate alcohol solution and the leg was draped. The initial approach was re-used. Pus samples were collected immediately after the capsule was breached and stored on ice until analyzed. The joint was then thoroughly debrided, a synovectomy was performed, and the joint space was irrigated with 200 ml of sterile saline using a 50 ml syringe. The wound closure was performed in a similar way to the first surgery. Following skin closure a dressing similar to the first surgery was applied.

#### Post-operative follow-up (incl. blood sampling and weight)

The pain level of the animals was monitored daily using the rabbit grimace scale (31). To further assess the well-being of the animals, the rabbits were weighed twice a week.

Blood samples were obtained every 2 days by venipuncture of the marginal ear veins to assess the evolution of the total leucocyte count, differential leukocyte count, c-reactive protein (CRP) serum levels, procalcitonin serum levels, and serum vancomycin levels if applicable. Samples were collected in either K3 EDTA tubes or gel and clot activator tubes (Vacutest Kima s.r.l., Arzergrande, Italy). Samples collected in K3 EDTA tubes were stored at 4°C before analysis. Samples collected in gel and clot activator tubes were kept at room temperature until complete coagulation and were then centrifuged at 2000 *g* for 10 min at 20°C. The serum was then collected and stored at –80°C before analysis.

#### Vancomycin treatment and pharmacokinetics

Vancomycin injections were initiated 1 week after surgery in group A (non-infected animals), and immediately after the debridement procedure in groups C. The animals of these two groups received 50 mg/kg of vancomycin by intra-venous injections through a catheter every 12 h for 7 days. This dosage was chosen following a literature review and its adequacy to attain therapeutic concentrations. Blood samples were obtained in group A animals before injection, and 1, 2, 4, 6, 10, and 12 h after the 1st and 5th doses to study the vancomycin serum concentration kinetics and confirm the adequacy of this regimen. Based on these results, the vancomycin serum concentrations in group C were monitored every 2 days 6 h after injection.

#### Euthanasia

All animals were euthanized 2 weeks after the initial surgery by an intravenous injection of 100 mg/kg of sodium pentobarbital (Dolethal, Vetoquinol, Magny-Vernois, France). The death of the animals was confirmed by cardiac auscultation. Following the euthanasia, the animals were installed in a Class II biosafety cabinet, the skin of the operated leg was disinfected twice with 2% chlorhexidine digluconate alcohol solution and the limb was draped. The knee was dissected in aseptic conditions. Pus, articular capsule, tibial bone, and the implant were sampled for microbiological analysis using clean instruments, rinsed in sterile PBS (except pus), fragmented with clean instruments (bone and capsule), transferred to sterile tared tubes containing five 3 mm steel beads (bone and capsule), and kept on ice before analysis. The anterior half of the tibial epiphysis and metaphysis were cut and were fixated in 4% buffered formaldehyde (VWR Chemicals, Radnor, PA, USA) for histological analysis.

### Colony-forming units counts

Bone and capsule samples were weighted, 3 ml of sterile PBS was added to the tubes and then homogenized by shaking for 15 min using a TissueLyser II device (Qiagen, Hilden, Germany). The tubes containing the implants were filled with 2 ml of sterile PBS, vortexed for 30 s, sonicated for 5 min (Branson 5510 Ultrasonic bath, Emerson Electric, Saint-Louis, MO, USA), and vortexed for 30 s. The pus samples were weighted, then 3 ml of sterile PBS was added to the tubes and the samples were vortexed for 1 min. Three aliquots of the recovered liquids from all samples were then serially diluted in sterile PBS and plated on TSA plates. The plates were incubated at 37°C for 7 days. Initial CFU counts were done after 48 h. The plates were examined after 7 days to identify possible contaminations.

### Serum antibiotic dosing

Vancomycin serum concentrations were quantified using the Roche VANC3 assay using a COBAS 8000 automated analyzer (Roche Diagnostics GmbH, Mannheim, Germany) on serum samples.

### Serum markers

CRP serum concentrations were assayed using the ABCAM rabbit CRP ELISA kit (ab157726; ABCAM, Cambridge, UK) on serum samples diluted 2.000–4.000 times in the supplied dilution solution, following the manufacturer’s instructions. Procalcitonin serum concentrations were assayed using the MyBioSource rabbit procalcitonin ELISA kit (MBS7606572; MyBioSource Inc., San Diego, CA, USA) on serum samples diluted 4 times in the supplied dilution solution, following the manufacturer’s instructions.

Creatinine serum concentrations were measured in group A animals using a Fuji Dri-Chem analyzer (Fujifilm Corporation, Tokyo, Japan) with CRE-PIII slides, as per the manufacturer’s instructions.

### Blood cells count

Manual counting methods were used for the leucocytes and differential counts. For leukocyte counts, blood samples collected in K3-EDTA tubes were diluted 20 times in Türk’s solution (3% acetic acid, 0.01% crystal violet, in mQ water). After 5 min, 10 μl of the diluted samples were placed in an improved Neubauer cell count chamber (Paul Marienfeld GmbH & Co. KG, Lauda-Königshofen, Germany). The cells in 4 large squares were counted and averaged. Three separate counts were performed for each sample. For differential counts, blood films were done from K3-EDTA blood samples. After fixation in methanol, the blood films were stained using the May-Grünwald-Giemsa technique (32). White blood cells were counted at 100× magnification to determine the differential counts.

### Histology

The tibial and femur samples were fixated in 4% buffered formaldehyde for 1 week. The samples were then decalcified in hydrochloric acid (DC3 decalcifier, VWR Chemicals) for 1 week and included in paraffin. A total of 5 μm sections were cut in a coronal plane using a Microm HM340 E microtome (Microm International GmbH, Walldorf, Germany) and mounted on SuperFrost Plus slides (Thermo Scientific, Waltham, MA, USA). Samples were stained with Masson’s trichrome blue stain. The slides were then scanned at a 200× magnification using SCN400 (Leica Biosystems GmbH, Wetzlar, Germany) scanner and examined for the qualitative assessment of inflammatory infiltration, and cartilage alterations.

### Data transformation and statistical analysis

CFU counts were transformed to base 10 logarithms and divided by the weight of the sample. The neutrophils over lymphocytes ratio was used to describe the differential counts. Hyperbolic interpolation was used to transform the raw ELISA results of serum CRP and procalcitonin assays. Vancomycin serum AUCs were computed using the trapezoid rule. The values reported in the text are the mean and the 95% CI (in brackets) or the median and the 95% CI obtained by bootstrapping (in brackets). The statistical analyses were done using GraphPad 8.0.1 (GraphPad Software Inc., San Diego, CA, USA) or R 3.6.2 [R Core Team, (33)].

## Results

### Post-operative outcomes

All animals survived until the end of the experiments, without reaching the predefined humane endpoints. Pain levels remained acceptable following the surgery. No wound discharge or opening was observed. Progressive weight loss was observed in animals of all groups and was more severe in groups B and C (Supplementary Figure 1). No implant loosening was observed during either debridement procedures or dissections.

### Vancomycin pharmacokinetics and therapeutic monitoring

**FIGURE 3:**
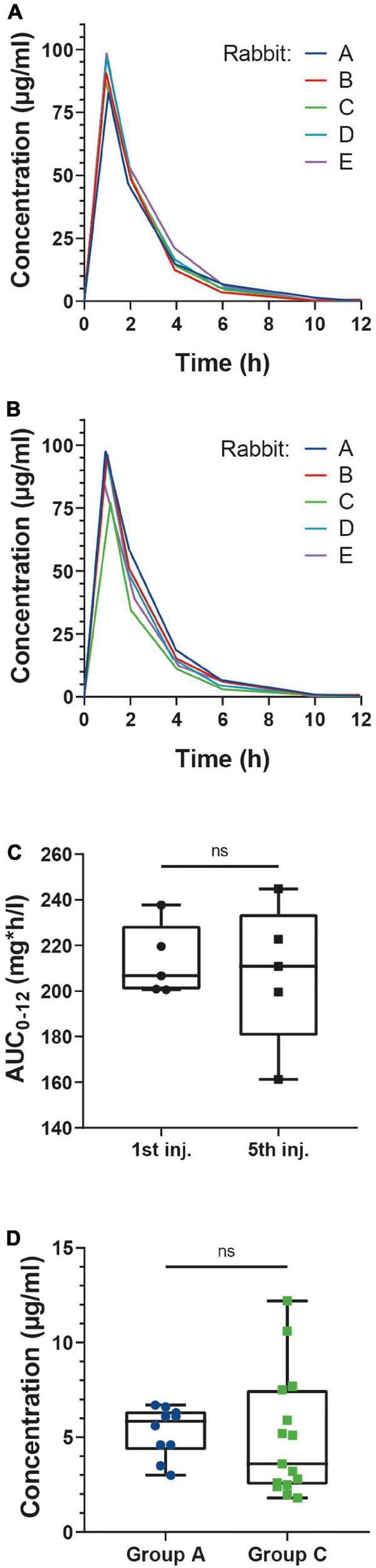
Serum concentration (mg/L) of vancomycin evolution following the first **(A)** and fifth **(B)** injections in group A rabbits. **(C)** Box plots of the AUC_0–12 h_ after the first (left) and fifth (right) injections. AUC_0–12 h_ were computed using the trapezoidal method. AUC_0–12 h_ were compared with the Wilcoxon test. *N* = 5. **(D)** Box plots of the serum concentrations of vancomycin 6 h after injection in groups A and C. Concentrations were compared with the Welch corrected *t*-test following normality evaluation using the Kolmogorov Smirnov test. *N* = 10 (group A), 15 (groups C).

The AUC_0–12 h_ were calculated in group A (non-infected animals). The mean AUC_0–12 h_ following the first vancomycin injection was 213.0 mg*h/L (SD: 15.79) (Figure 3A). Following the fifth injection, the median AUC_0–12 h_ was 207.8 mg*h/L (SD: 30.95) (Figure 3B). The comparison of the two AUC_0–12 h_ using the Wilcoxon signed-rank test showed no statistically significant difference (*p* = 0.81, Figure 3C).

Serum creatinine concentrations in group A animals were found to be stable throughout the experiment, with no influence of either the surgery or the administration of vancomycin (Supplementary Figure 2).

As the vancomycin serum concentrations approached 0 μg/ml 12 h after the injections, we decided to perform the therapeutic monitoring of group C animals 6 h after the vancomycin injections. The comparison of the 6 h vancomycin serum concentrations of groups A and C using the Kruskal-Wallis test showed no statistically significant differences (*p* = 0.55, Figure 3D).

### Microbiology

**FIGURE 4:**
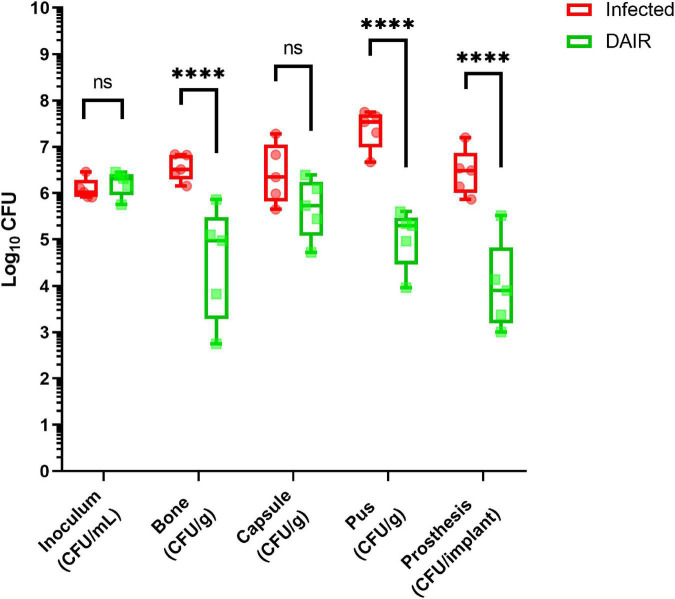
Bacterial loads in the different samples for rabbits of groups B (red) and C (green), and multiple comparisons. Statistical analysis: two-way ANOVA and Tukey HSD. ^ns^*p* > 0.05; *p < 0.05; ^**^p < 0.01; ^***^p < 0.001; ^****^p < 0.0001. *N* = 5.

CFU counts for all conditions are illustrated in Figure 4. No bacterial contamination of group A samples was observed, confirming the aseptic character of the surgical procedures. The bacterial concentrations of the inoculums were similar in all groups (∼6.5 log10 CFU/ml, *p* = 0.99), with an effective inoculum of ∼5.5 log10. At the time of debridement, the CFU counts in the pus of group C were of 7.2 log10 CFU/g (SD: 0.4). After euthanasia, we observed reproducible CFU counts in all samples of group B animals. Statistically significant reductions in CFU counts were observed in bone, pus, and implant samples of group C compared to group B (*p* < 0.0001), albeit with more variability. No statistically significant reduction was observed in capsule samples despite a reduced average (mean difference = 0.74 log10 CFU/g, *p* = 0.35).

### Serum markers

**FIGURE 5:**
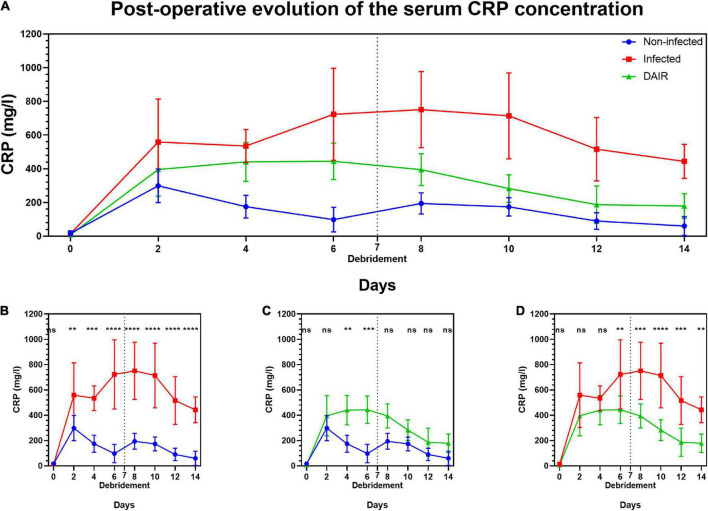
Post-operative evolution of the serum C-reactive protein (mg/l) in groups A, B, and C **(A)** and comparisons between groups A and B **(B)**, A and C **(C)**, B and C **(D)**. Vertical dashed lines indicate the day of debridement surgery. Statistical analysis: two-way repeated-measures ANOVA and Tukey HSD *post hoc* test. ^ns^*p* > 0.05; *p < 0.05; **p < 0.01; ***p < 0.001; ****p < 0.0001. *N* = 5.

Serum CRP concentrations are illustrated in Figure 5. Baseline CRP serum concentrations were found to be similar across the groups. The CRP levels increased rapidly in infected animals and a statistically significant difference with group A animals was observed from day 2 (group B) or day 4 (groups C and D). The evolution of serum CRP concentrations of groups B and C was similar until day 4, after which the serum CRP concentrations were significantly lower in group C. The CRP concentrations of group C animals steadily decreased following the debridement surgery and were no longer found to be statistically different from group A animals after day 8 (group C).

Procalcitonin serum concentrations were found to fluctuate throughout the experiment, with no influence of group allocation (Supplementary Figure 3).

### Leukocytes counts

**FIGURE 6:**
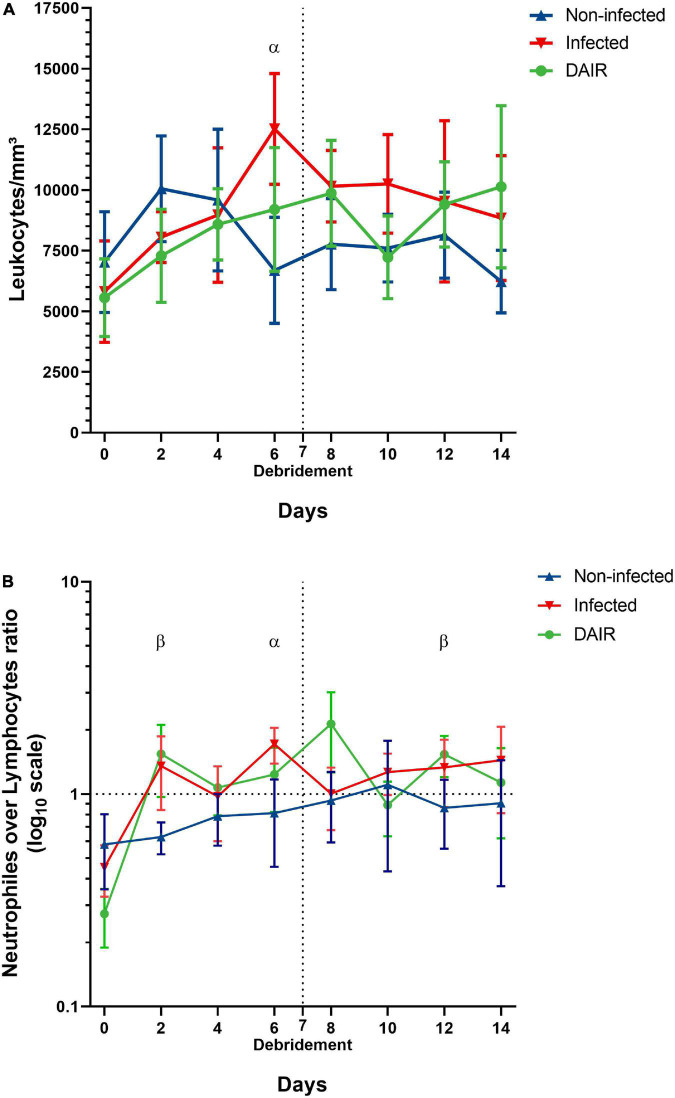
Post-operative evolution of the total leukocytes counts **(A)** and neutrophils over lymphocytes ratio expressed in base 10 logarithms **(B)**, blue: Group A (non-infected animals); red: Group B (infected animals); green: Group C (animals treated with DAIR). Vertical dashed lines indicate the day of debridement surgery. Horizontal dashed lines indicate an N/L ratio of 1. Statistical analysis: two-way repeated-measures ANOVA and Bonferroni *post hoc* test. α: statistically significant difference between groups A and B; β: statistically significant difference between groups A and C. *N* = 5.

Leukocytes counts are shown in Figure 6 panel A. The baseline leukocytes counts were similar across the three groups and a uniform increase was observed following the initial surgery. A return to pre-operative levels was observed in group A animals 6 days after the initial surgery, while a sustained increase was observed in the other two groups, with significant differences with group A observed at day 6 (group B, *p* = 0.008). The peak in leukocytes counts in group C was observed at day 8. No differences were observed between the three groups after day 8.

Neutrophils over lymphocytes ratios are shown in Figure 6 panel B. No significant differences were observed at baseline. A sharp increase in the ratios was observed in infected groups following the initial surgery, diverging clearly from group A animals at day 2, and was sustained during most of the experiment. Statistically significant differences were observed between groups A and C at days 2 (*p* = 0.048) and 12 (*p* = 0.026), and between groups A and B at day 6 (*p* = 0.008). The N/L ratio in group C animals peaked at day 8 but was not statistically different from other groups.

### Histology

**FIGURE 7:**
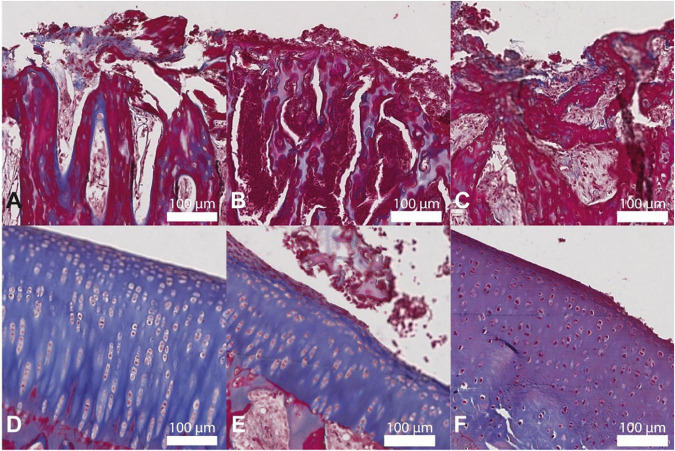
Histological images of the bone-implant interface and articular cartilage stained with Masson’s blue trichrome of groups A (panels **A,D**, respectively), B **(B,E)**, and C **(C,F)**. The infiltrates observed in the bone trabeculae in panel **B** are absent in panel **A** and are greatly reduced in panel **C**. The cartilage surfaces in panels **D–F** are similar. A total of 200× magnification, scale bar 100 μm.

The histological examination of the samples colored with Masson’s blue trichrome (Figure 7) showed that the bone trabeculae in direct contact with the implant were abundantly infiltrated with granulomatous materials. Granulomas were also observed on the surface of the cartilage and in articular recesses. These infiltrates were absent in group A animals and were significantly reduced in groups C animals. The articular cartilage surfaces appeared healthy in all three groups. Minimal cartilage ulcerations were observed in limited cases and did not appear to be associated with a particular group.

## Discussion

Our study describes the development of a novel *in vivo* model of acute PJI using custom-designed 3D printed Ti-6Al-4V unicompartmental tibial implants, infected per-operatively with a reference MRSA strain and treated with DAIR. This model allowed us to reproducibly reach the usual therapeutic targets for the selected antibiotic (vancomycin) and to monitor the effect of the treatment in infected animals treated with DAIR.

This model presents significant advantages and improvements over others previously described, making it especially suited for the study of PJI treated with DAIR.

The choice of a unicompartmental tibial implant 3D printed in Ti-6Al-4V implanted in a weight-bearing position, exposed to friction, and accessible to a mechanical debridement increases the clinical relevancy over earlier models.

Indeed, most models use non-clinically relevant implants, using either k-wires implanted in small rodents femurs – thus not exposed to the joint space or to the mechanical constraints usually applied to arthroplasty implants and inaccessible to a surgical debridement – (28, 34–37), screws implanted in the lateral femoral condyles of rabbits – thus not exposed to mechanical stresses – (38–40), or the use of silicone elastomer implants replacing the proximal part of the tibia in rabbits but made of a material of no clinical relevance to PJI (41).

The exposure of the implants to mechanical stresses and the choice of clinically relevant materials are crucial considerations for the design of models used to study infections where biofilm formation is expected. Mechanical stresses such as shear stress and pressure are known factors driving the production of extracellular polymeric substance in *S. aureus* biofilms and inducing water flux within the biofilms (42, 43). Also, the adhesion of bacteria and the development of biofilm have long been known to be strongly influenced by the composition (44, 45) and the surface properties of their substratum (46, 47). The use of other materials has been noted by previous authors as limiting clinical relevancy (26, 38).

Over the last decade, a trend toward the development of clinically relevant implants was observed, facilitated in part by the advances in additive manufacturing (27, 30, 48). These models are characterized by the use of load-bearing knee replacement implants manufactured using clinically relevant materials such as stainless steel, titanium alloy, and polyethylene. However, most of these models present limitations in their experimental and clinical relevance for the study of DAIR inherent to the choice of animal species.

In a rat model of total knee arthroplasty infected with different *S. aureus* inoculums, the authors observed a normalization of the inflammation parameters after 2 weeks in the absence of treatment. This spontaneous healing was attributed to a strong immune response of the rats and was considered by the authors to be a strong limitation to the use of the model for the study of PJI treatments (48).

Carli A. et al. reported the first use of 3D printed implants in a PJI model in 2016 (26). The authors designed a Ti-6Al-4V tibial replacement implant adapted to mice knees and infected by the intra-articular injection of *S. aureus* (30). This model was recently used to study the application of an adjuvant treatment using bacteriophage lysins after a surgical debridement followed by a vancomycin treatment (49). While this model reproduces PJI in a clinically relevant manner, the use of mice presents intrinsic limitations. The modest size of mice knees limits the possibilities of sampling for analysis, requiring the use of multiple animals to study the bacterial load and the histological alterations of the bone. Moreover, the poor venous access and limited blood volume of mice hiders the reproduction of clinically relevant antibiotics pharmacokinetics and their monitoring as well as the longitudinal study of the hemogram and inflammatory markers.

The choice of larger animals such as NZW rabbits allowed us to multiply the number of samples available from each specimen, facilitating the comparison of microbiological and histological samples, and the longitudinal monitoring of CRP, PCT, and leukocyte counts, while also enabling the administration of vancomycin by intravenous injections and its monitoring, reproducing a clinically relevant regimen.

Similar sampling considerations lead Lopez et al. to develop a comparable model of rabbit tibial arthroplasty using 3D printed implants infected by an intra-articular inoculation with *S. aureus* (27, 50). However, to our knowledge, this model has not been used yet to study therapeutic interventions following infection.

We used a 5.5 log_10_ CFU bacterial inoculum similar to what is usually reported in the literature (27–30), inducing infections in all animals with no fatal sepsis observed. These infections resulted in reproducible *S. aureus* counts in all samples at one- and 2 weeks post-surgery. The observed CFU counts are however difficult to compare to those of previous studies because of an important variability in protocols and the reporting of values.

Debridement surgery, followed by the intravenous administration of vancomycin, significantly reduced the bacterial counts in all samples, except for the joint capsule, despite a careful synovectomy. Following DAIR, the reductions in CFUs in bone samples and on the implants appeared to be proportionately more pronounced than what Sosa et al. reported (49). The increased impact of DAIR on CFU counts in our model may result from multiple factors, including a more thorough debridement procedure and a better-controlled vancomycin regimen.

Indeed, we observed that the intravenous injection of 50 mg/kg of vancomycin twice a day consistently achieved the commonly accepted therapeutic target for the treatment of *S. aureus* infections of an AUC_0–24 h_ over MIC ratio over 350 (51) in non-infected animals. No renal toxicity was observed using this dosing regimen. We were able to monitor the vancomycin serum concentrations in infected animals and observed no significant discrepancy with the non-infected control group. Previously published studies investigating the pharmacokinetics of vancomycin in NZW rabbits found that similar dosing regimens were required to reach this target (52, 53). In contrast to these studies, we observed higher peak concentrations and lower trough concentrations, suggesting a higher clearance in our model. These lower through concentrations drove us to monitor the vancomycin serum concentrations of the infected animals 6 h after the injection instead of the usual through concentration monitoring.

Our model presents some limitations. The most significant is the limited tolerance of rabbits to some antibiotics, including rifampin. Gatin et al. reported a 23% fatality rate in a rabbit PJI model when the treatments included rifampin (54). Rifampin, in association with other antibiotics, is known to be effective against staphylococcal biofilms *in vitro* and *in vivo* (55), and its use in combination with another antibiotic in the treatment of patients suffering from staphylococcal PJI is recommended by the IDSA (56). Still, as our model was designed to evaluate adjuvant treatments to DAIR, we believe that the use of a suboptimal antibiotic therapy may be justified to emphasize the effect of the tested agent.

The timing for intervention was arbitrarily defined to fit the usual definition of an acute PJI while maintaining clinical relevancy and experimental practicality by limiting the incubation period to 7 days. While the study of variable intervals between inoculation and therapeutic interventions could provide additional information, these supplemental experiments were out of the scope of this preliminary study. Similarly, while the addition of an additional group of animals treated only with surgery would have provided an additional control, we considered that it would be beyond the focus of the current work where we strived to reproduce clinically relevant conditions.

Infected animals gradually lost weight throughout the experiment, approaching the predefined humane endpoints 2 weeks after inoculation. This limits the utilization of our model to short treatment periods. The use of lower inoculum could be considered but carries the risk of not inducing infections in all animals (48).

While no implant loosening was observed, the absence of bone cement means that the bone cut may have been incompletely covered by the implant. However, as radiolucent lines are common following joint replacement (57), we believe this does not impact the relevancy of our model. Moreover, this could prove advantageous to show the impact of injectable treatments of biofilms by allowing the formation of biofilm inaccessible to mechanical debridement.

The development of biofilm on the surface of the implants was not investigated in these experiments as it was not part of the study protocol submitted to the ethical comity and would have required the allocation of additional animals and implants to this effect. Indeed, the methods for the identification of biofilms on implants most often rely on fluorescence microscopy (58) or scanning electron microscopy (30, 59, 60), techniques incompatible with the initial study goal of determining the bacterial load on implants. Further experiments could include separate groups or additional implants to study biofilm development on implants.

The study was limited to the use of a single reference MRSA strain, using a set inoculum of 5.5 log_10_ CFU. While common in the literature for *in vivo* models, the use of clinical strains may have given greater relevance to our study. However, the use of a widely available reference strain carries a few advantages. The strain is easily available facilitating the replication of our experiments, its genome has been sequenced and is readily available, does not carry the *pvl* gene, limiting the virulence of the infection, and has been used in multiple previous biofilm experiments in our laboratory, facilitating the comparison of our *in vitro* and *in vivo* works. A set inoculum, chosen after a literature review (27–30), was preferred over repeated experiments comparing varying bacterial loads in order to reduce the total amount of animals used to conform to ethical standards of animal use reduction.

Despite these limitations, the clinical relevancy and the reproducibility of this model make it a prime choice to study DAIR and adjuvant therapies applied in this context. Further refinements could be brought by studying the use of clinical strains or of other bacterial species found in PJI, as well as by investigating the pharmacokinetics and tolerance of other antibiotic agents.

## Conclusion

This study describes the development of a novel clinically relevant *in vivo* model of acute MRSA prosthetic joint infection in New Zealand White rabbits treated with surgical debridement and an intra-venous vancomycin treatment. We showed that this model allowed for the development of a reproducible infection in all subjects. Vancomycin was administered intravenously twice a day, using a dosing regimen that attained the clinical targets for *S. aureus* infections and combined with a thorough debridement procedure, significantly reduced the bacterial load in most samples. The reproducibility and clinical relevancy of our model could make it well suited for the study of innovative adjuvant treatments against PJI such as bacteriophages combinations or biofilm targeting hydrolytic enzymes.

## Data availability statement

The raw data supporting the conclusions of this article will be made available by the authors, without undue reservation.

## Ethics statement

This animal study was reviewed and approved by the Commission d’Ethique pour l’Expérimentation Animale Secteur des Sciences de la Santé Université catholique de Louvain (2019/UCL/MD/041).

## Authors contributions

HP designed and performed the experiments, analyzed the data, and drafted the manuscript. All authors contributed to the revision and improvement of the manuscript.
