# Supplementary material for: Development of an innovative in vivo model of PJI treated with DAIR
Source: Front Med (Lausanne). 2022 Oct 13;9:984814. doi: 10.3389/fmed.2022.984814 (PMC9606572; doi:10.3389/fmed.2022.984814)
Supplement: Supplementary file 1 [file Data_Sheet_1.pdf]

1 Supplementary material

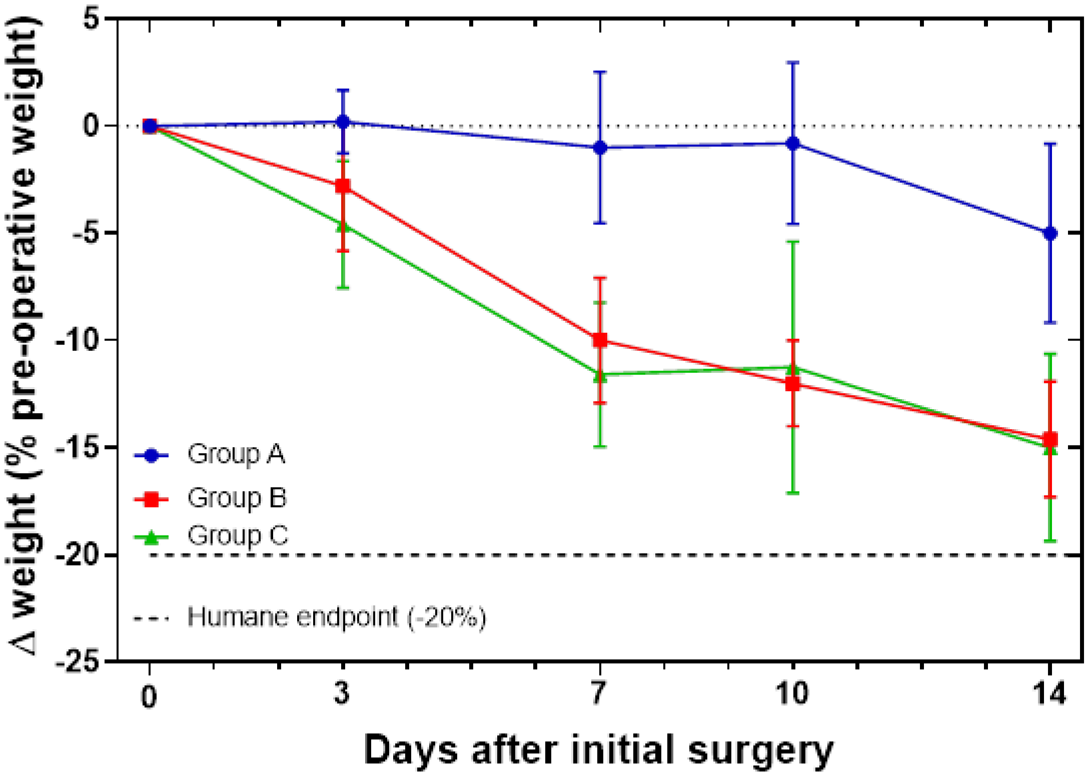

Supplementary figure 1. Post-operative evolution of the animals' weight, expressed as a percentage of the pre-operative weight.

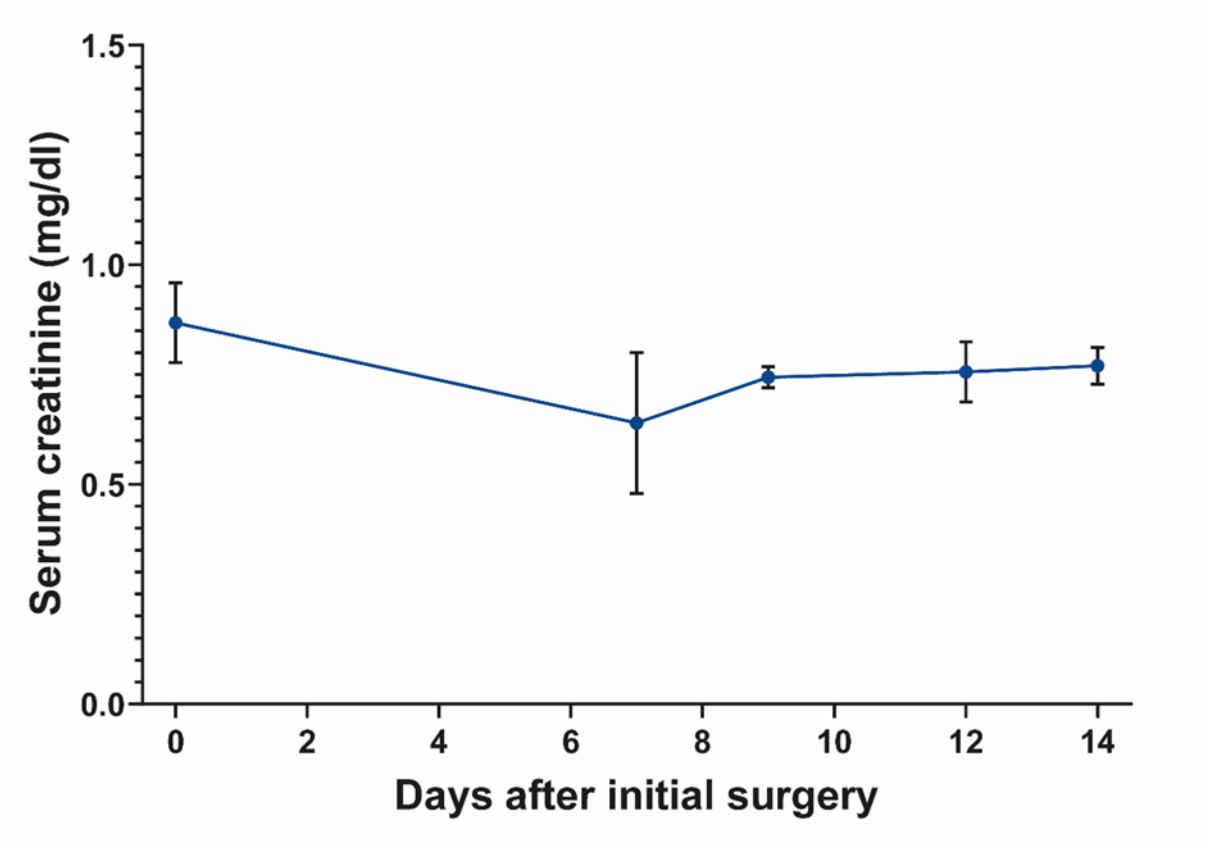

Supplementary figure 2. Post-operative evolution of group A animals' serum creatinine concentration.

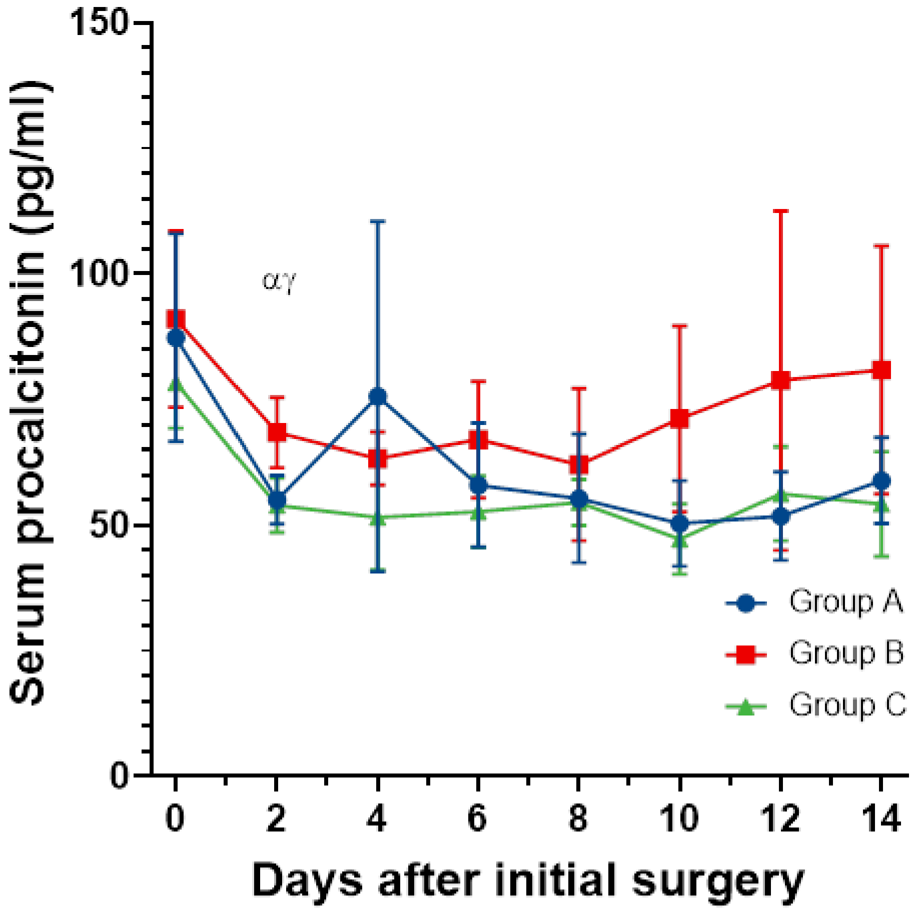

Supplementary figure 3. Post-operative evolution of the serum procalcitonin concentrations (pg/ml). Statistical analysis: two-way repeated-measures ANOVA and Tukey post-hoc test. α: statistically significant difference between groups A and B; γ: statistically significant difference between groups B and C. N = 5.
